# Supplementary material for: Effects of Genetic Variation of the Sorting Nexin 29 (SNX29) Gene on Growth Traits of Xiangdong Black Goat
Source: Animals (Basel). 2022 Dec 8;12(24):3461. doi: 10.3390/ani12243461 (PMC9774745; doi:10.3390/ani12243461)
Supplement: Supplementary file 1 [file animals-12-03461-s001.zip › 1-Supplementary Table.pdf]

Supplementary Table:

Table S1. The information of primers used in this study.

	Gene	Primer sequences (5'to 3')	Tm(℃)	Product length (bp)
DNA level	*SNX29*-CNV	F:AGGTGACATCGGTGCTCAGTGT	61	107
		R: GCCTGCTCCTGGAAGAGAATG		
	MC1R	F: GGGCAGTCCCTTGACAAAGA	58	129
		R: ATCTCCCCAGCCTCCTCATT		
	Indel-17bp	F: TGGGCACATACATTTCAGGAC	56	144
		R: AAGCTCCCAAAGTCAGGAACC		
	Indel-21bp	F:GAGGAGATAAAGGGAGGGAGTC	56	199
		R: AGAAAGGCAGGCAGGTAAGG		
mRNA level	*SNX29*	F: GAACCTGCTTGACGGCGAGATG	59	87
		R: TCCTCCTGTTCAGCCACCTTCC		
	GAPDH	F: AAAGTGGACATCGTCGCCAT	57	116
		R: CCGTTCTCTGCCTTGACTGT		

Table S2. Genotypic and allelic frequencies and population indexes of *SNX29* gene in Xiangdong black goats.

Loci	Total	Genotypes	Genotypic frequencies	Allelic frequencies	HWE *P* values	Ho	He	Ne	PIC
17bp-	516	II (360)	0.698	I (0.847)	*P*>0.05	0.740	0.262	1.351	0.227
		ID (153)	0.297	D (0.155)					
		DD (3)	0.006						
21bp-	516	II (183)	0.355	I (0.565)	*P*>0.05	0.509	0.493	1.964	0.371
		ID (216)	0.419	D (0.437)					
		DD (117)	0.227						

Note: II: homozygote insertion genotype; DD: homozygote deletion genotype; ID: heterozygote genotype; HWE: Hardy–Weinberg equilibrium; Ho: homozygosity; He: heterozygosity; Ne: effective allele numbers; PIC: polymorphism information content.

Table S3. Relationship between the indel loci within *SNX29* gene and growth traits in Xiangdong black goats.

		**Observed genotypes (Mean ± SE)**			
**Loci**	**Growth traits**	**II (n=360)**	**ID (n=153)**	**DD (n=3)**	***P* Values**
17bp-	Body height (BH)	56.58±0.42	56.04±0.66	60.00±2.00	0.412
	Body length (BL)	58.56±0.53	59.12±0.71	61.50±0.50	0.641
	Body oblique length (OL)	58.81±0.53	59.82±0.71	60.5±2.50	0.493
	Chest	73.71±0.71	72.27±1.24	75.5±0.50	0.521

	circumference (CC)				
	Abdominal circumference (AC)	84.64±0.96	84.02±1.41	82.00±4.00	0.884
	Cross height (CH)	57.50±0.43	57.14±0.50	59.00±0.00	0.777
	Cannon Circumference (CAC)	10.81±0.12	10.92±0.16	12.00±0.00	0.376
	**Growth traits**	**II (n=183)**	**ID (n=216)**	**DD (n=117)**	***P* Values**
21bp-	Body height (BH)	56.56±0.58	56.44±0.61	56.36±0.64	0.985
	Body length (BL)	58.78±0.75	58.73±0.65	58.72±0.84	0.98
	Body oblique length (OL)	59.81±0.69	58.84±0.67	58.64±0.77	0.476
	Chest circumference (CC)	73.48±1.07	72.8±0.94	73.77±1.30	0.818
	Abdominal circumference (AC)	85.38±1.42	83.27±1.19	84.85±1.56	0.496
	Cross height (CH)	57.47±0.53	56.94±0.59	58.08±0.57	0.414
	Cannon Circumference (CAC)	$10.91±0.17^{ab}$	$10.73±0.15^{b}$	$10.95±0.17^{ab}$	0.626

Note：Data were shown as mean ± SE.
