# Supplementary material for: Effects of Genetic Variation of the Sorting Nexin 29 (SNX29) Gene on Growth Traits of Xiangdong Black Goat
Source: Animals (Basel). 2022 Dec 8;12(24):3461. doi: 10.3390/ani12243461 (PMC9774745; doi:10.3390/ani12243461)
Supplement: Supplementary file 1 [file animals-12-03461-s001.zip › 4-Supplementary Figures.pdf]

Supplementary Figures

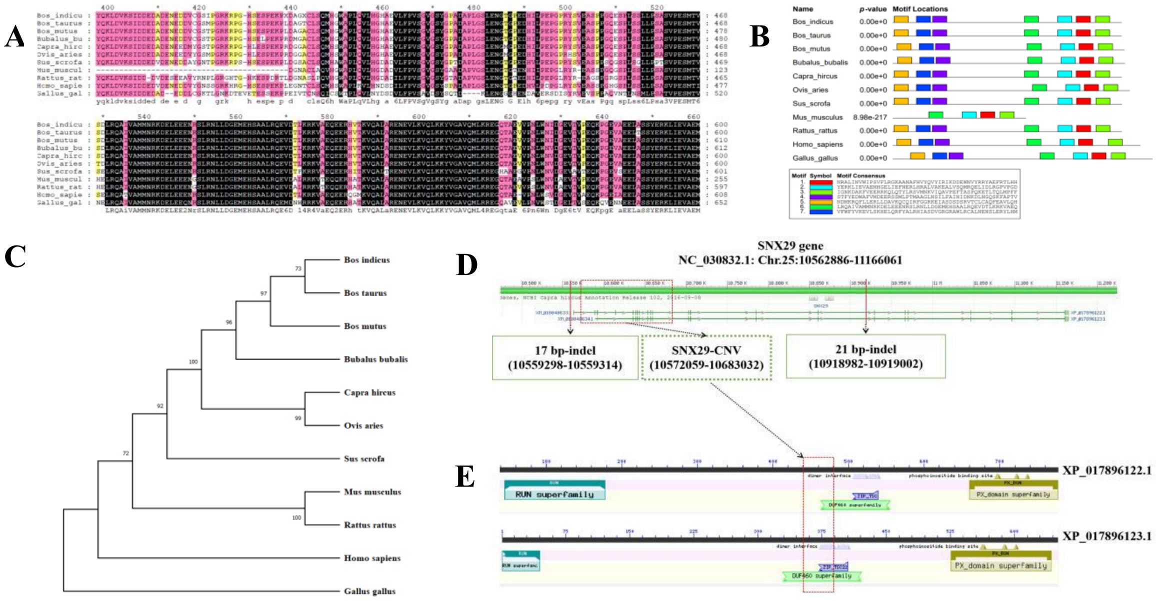

Figure S1. Biological evolution, CNV and Indels loci and the related conserved domains of SNX29. (A) Multiple sequence alignment of SNX29 for the 11 species. The degree of similarity is delineated using different background shading, with black 100%, pink 80%, yellow 60%, and white not conserved. (B) Motif structural analysis for SNX29 among 11 species. The length of the color block shows the location, intensity and importance of a particular motif position. (C) Phylogenetic tree analysis for SNX29 gene among 11 species. The amino acid sequences of the SNX29 protein for the 11 species were downloaded from the NCBI database (https://www.ncbi.nlm.nih.gov/protein). Phylogenetic tree analysis for SNX29 protein among different species were performed by MUSCLE neighbor-joining (NJ) method with MEGA X 10.2.6 software. (D) The location of *SNX29* CNV and two indels in goat *SNX29* gene DNA region and mRNA region, respectively based on NC_030832.1 and XM_018040633.1/XM_018040634.1. For the two indels, the 17bp indel (g.10559298-10559314) sequence was located in the upstream of the *SNX29* gene and the 21bp indel (g.10918982-10919002) sequence was located in the intron region. *SNX29* CNV was overlapped with several extrons and introns of *SNX29* gene. (E) The schematic diagram of overlaps between SNX29 *CNV* - related protein region and conserved domains in SNX29 protein sequence (XP_017896122.1/XP_017896123.1) based on NCBI CDD. Each color bar is a specific hit representing a different domain superfamily.

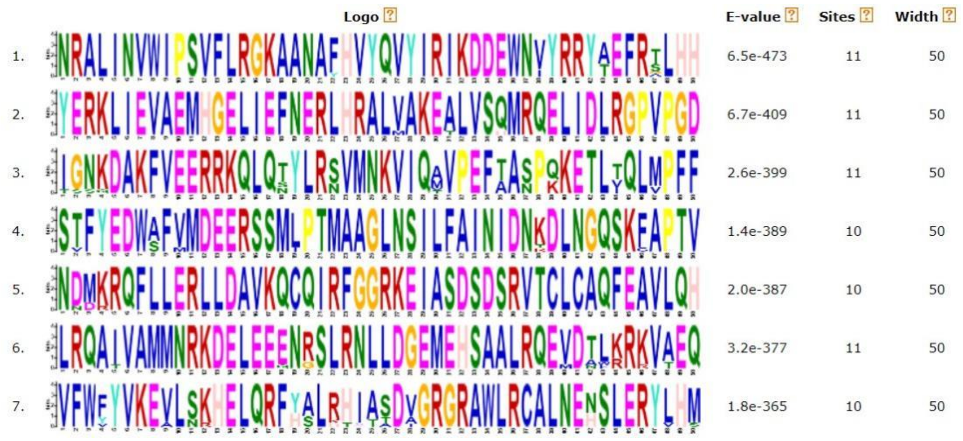

Figure S2. Significant motifs of *SNX29* across the eleven species. Motifs were detected using the MEME suite. The different color letters show abbreviations of different amino acids, given through motif analysis on the MEME suite system.

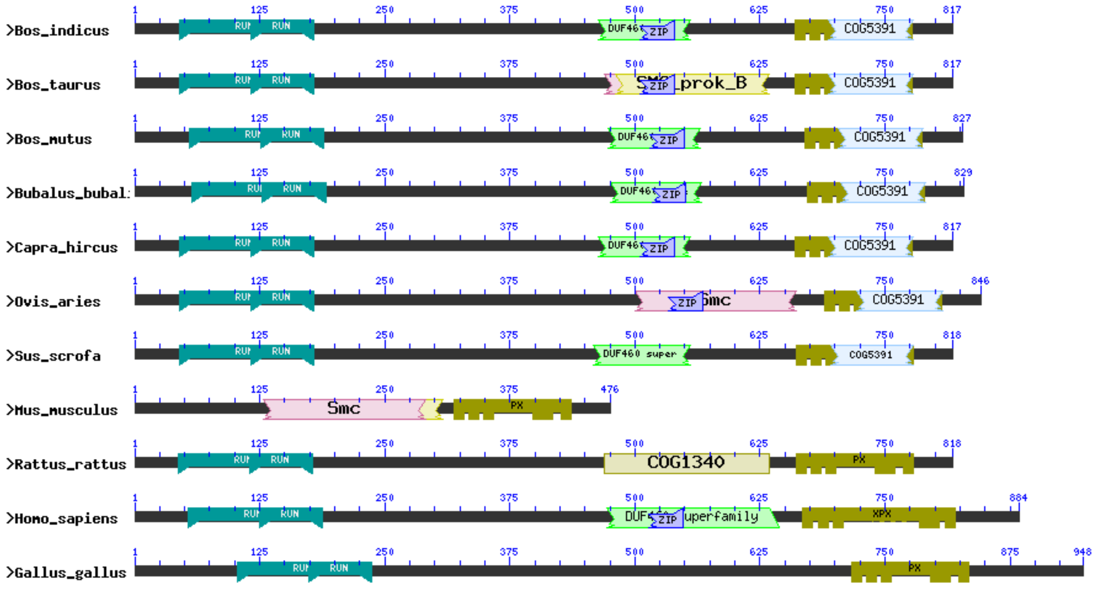

Figure S3. Structure of *SNX29* protein domain families in the eleven species. Each color bar is a specific hit representing a different domain superfamily.

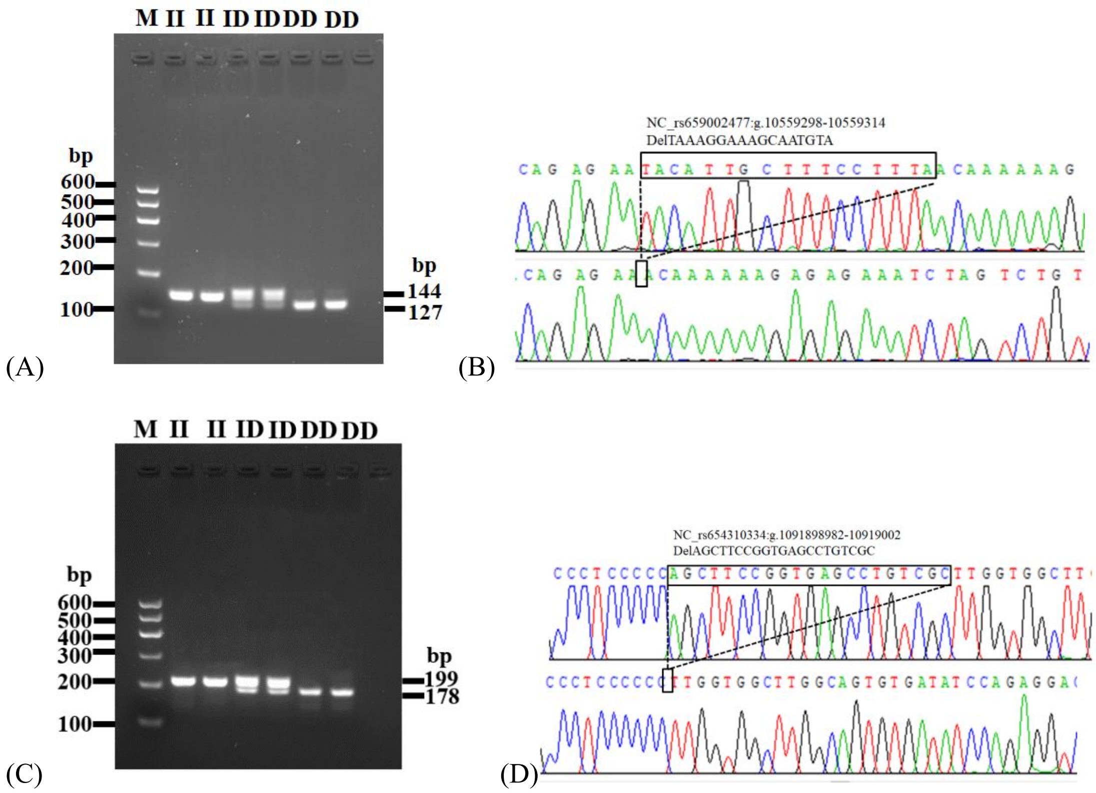

Figure S4. Agarose electrophoresis and sequence chromatograms of two indel loci within goat *SNX29* gene. II, homozygote insertion genotype; DD, homozygote deletion genotype; ID, heterozygote genotype. (A) Genotyping of 17 bp indel. ID genotype is represented by the combination of 144 and 127 bp bands; II genotype is represented by 144 bp band; DD genotype is represented by 127 bp band. (B) Sequencing maps for the 17 bp indel within the goat *SNX29* gene. (C) Genotyping of 21 bp indel. ID genotype is represented by the combination of 199 and 178 bp bands; II genotype is represented by 199 bp band; DD genotype is represented by 178 bp band. (D) Sequencing maps for the 21 bp indel within the goat *SNX29* gene.

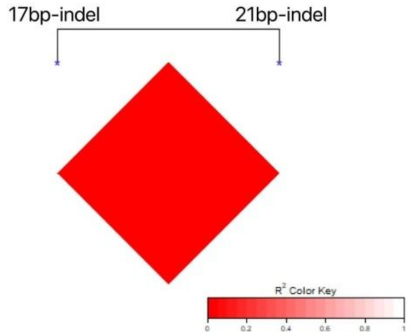

Figure S5. LD heatmap of 17bp indel and 21bp indel within goat *SNX29* gene.
