# Supplementary figures and images for: Effects of Genetic Variation of the Sorting Nexin 29 (SNX29) Gene on Growth Traits of Xiangdong Black Goat
Source: Animals (Basel). 2022 Dec 8;12(24):3461. doi: 10.3390/ani12243461 (PMC9774745; doi:10.3390/ani12243461)

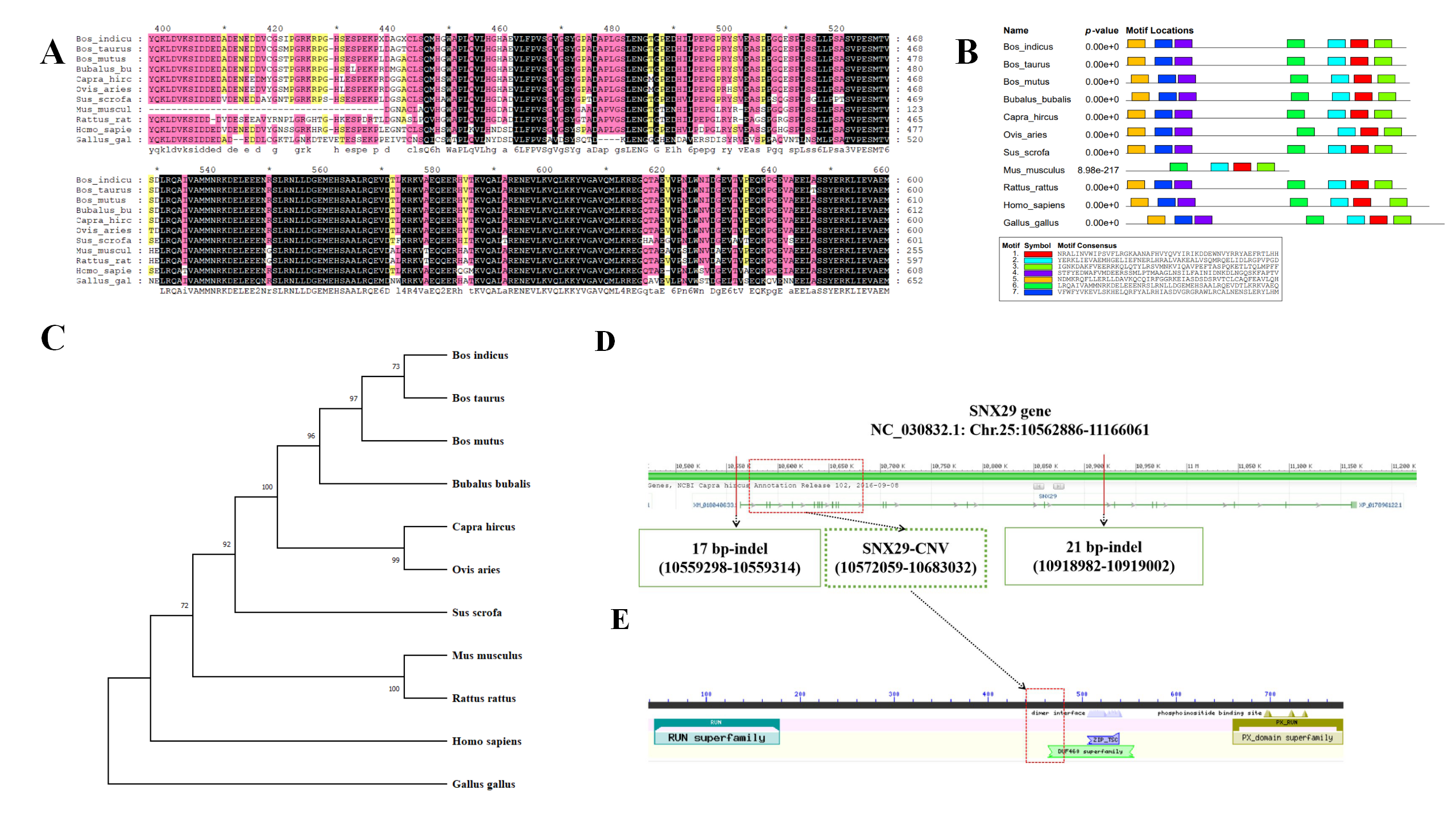

Supplement: Supplementary file 1 [file animals-12-03461-s001.zip › FIGURE S1.png]
